# The characteristics and clinical relevance of tumor fusion burden in head and neck squamous cell carcinoma

**DOI:** 10.1002/cam4.4890

**Published:** 2022-05-27

**Authors:** Lirui He, Dandan Ren, Guoqing Lv, Beibei Mao, Lijia Wu, Xiaoyu Liu, Longlong Gong, Ping Liu

**Affiliations:** ^1^ Department of Gastrointestinal Surgery Peking University Shenzhen Hospital Shenzhen China; ^2^ Genecast Biotechnology Co., Ltd Jiangsu Province China; ^3^ Department of Oncology The Second Xiangya Hospital of Central South University Changsha Hunan Province China

**Keywords:** head and neck squamous cell carcinoma, human papillomavirus, immune infiltration, tumor fusion burden

## Abstract

**Background:**

Recent studies suggest that tumor fusion burden (TFB) is a hallmark of immune infiltration in prostate cancer, the correlation of TFB with immune microenvironment, and genomic patterns in head and neck squamous cell carcinomas (HNSC) remain largely unclear.

**Methods:**

Gene fusion, genomic, transcriptomic, and clinical data of HNSC patients from the cancer genome atlas (TCGA) database were collected to analyze the correlation of TFB with mutation patterns, tumor immune microenvironment, and survival time in HNSC patients.

**Results:**

Human papillomavirus (HPV) (−) patients with low TFB exhibited significantly enhanced CD8+ T cells infiltration and cytolysis activity and increased level of interferon‐gamma (IL‐γ), human leukocyte antigen (HLA) class I, and chemokines. Moreover, TFB was positively correlated with TP53 mutation, score of gene copy number, and loss of heterozygosity (LOH), as well as the biological progress of epithelial‐mesenchymal transition (EMT), metastasis, and stem cell characteristics. Further analysis revealed that HPV (−) HNSC patients with low TFB have a better prognosis.

**Conclusions:**

Our data revealed the correlation of TFB with tumor immune microenvironment and predictive features for immunotherapy, implying tumors with low TFB may be potential candidates for immunotherapeutic agents. Moreover, the TFB low group had prolonged overall survival (OS) in the HPV (−) HNSC cohort.

## Graphical abstract

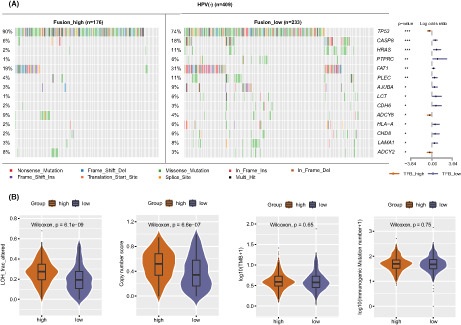

## 1 INTRODUCTION

Head and neck squamous cell carcinoma (HNSC) originates from the epithelial cells lining the mucosa of the upper respiratory tract and food passage.^1^ Due to the complex anatomical structure and a large number of genetic changes, HNSC is highly heterogeneous.^2^ Smoking and drinking are the main factors that trigger HNSC,^3^ and subsequent studies have shown that human papillomavirus (HPV) is closely related to HNSC, especially oropharyngeal squamous cell carcinomas(OPSCCs).^4^ The onset of HNSC is insidious, and most of the patients are already at an advanced stage at the first diagnosis. Although the treatment methods based on surgery, radiotherapy, and pure systemic treatment have been improved continuously, the survival rate of HNSC patients remains low, and the prognosis is poor.^1^ Interestingly, compared with human papillomavirus (HPV) (−) HNSC, HPV (+) HNSC patients are more effective for immunotherapy.^5^, ^6^ Hence, finding accurate and reliable biomarkers is particularly important for the optimization of stratification, treatment options, and prognosis prediction for HNSC patients.

Gene fusions occur in various cancers and can be used as cancer‐specific markers. Early research was focused on driver gene fusions for therapeutic purposes.^7^, ^8^ For example, crizotinib (ALK gene fusion)^7^ is a targeted drug for nonsmall‐cell lung cancer. Recent studies have shown that prostate cancer patients with high tumor fusion burden (TFB: the number of fusion genes per 10,000 genes) have a significant increase in the number of tumor‐infiltrating lymphocytes (TILs), indicating that TFB can affect the tumor immune microenvironment.^9^ In addition, gene fusion is also an ideal source of tumor neoantigens and plays an important role in current tumor immunotherapy, and fusion gene neoantigens are emerging targets for tumor immunotherapy and can be used for the development of tumor immune microenvironment regulation.^10^ Yang et al.^11^ found that gene fusion is the source of a case of transferred HNSC immunogenic neoantigen, which can mediate the response to immunotherapy. Extending this conclusion to other cancers, Hindson et al.^12^ found in 20 cases of head and neck adenoid cystic carcinoma that gene fusion antigens that can cause cytotoxic T cell responses were negatively correlated with immune cell infiltration. These findings have a certain understanding of the relationship between gene fusion and tumor immune microenvironment and have certain significance for cancer immunotherapy.

In this study, we analyzed the TFB characteristics of HNSC in the TCGA database. Furthermore, we divided the HNSC patients into HPV‐negative and HPV‐positive groups and investigated the gene mutation characteristics and immune cell distribution of the tumor microenvironment with regard to the TFB condition (high/low) as well as the impact on the survival of patients.

## 2 METHODS

### 2.1 Data source and processing

The gene expression data of HNSC cohort were downloaded from TCGA official website (https://portal.gdc.cancer.gov). The gene expression data of TCGA in the format of fragments per kilobase per million mapped reads (FPKM) were then converted to that transcripts per kilobase million (TPM) for subsequent analysis. The clinical data and single‐nucleotide variant (SNV) profiles were obtained from the cbioportal (https://www.cbioportal.org/). The clinical data include age, gender, HPV infection, race, tumor stage, and so forth (see “Detailed data of the study.xlsx”).

Gene Fusion data were collected from the published literature pertaining to TCGA cohort,^13^ using multiple RNA‐Seq fusion callers, namely STAR‐Fusion (https://github.com/STAR‐Fusion/STAR‐Fusion/wiki), EricScript (https://sites.google.com/site/bioericscript/), and BREAKFAST (https://github.com/annalam/breakfast). Then, strict filtering strategies were used to identify fusion gene events from the gene fusion data. TFB was defined as the number of fusion genes in each tumor. Patients with TFB >1 and those with TFB ≤ 1 were classified into the TFB high and TFB low groups, respectively.

The abundances of 28 immune cell types were calculated by using ssGSEA. The gene sets for HLA‐I (MHC‐class I) signature, cytolytic activity, immune costimulators, chemokines, immune inhibitors, the IFN‐γ signature, and MHC‐class II signature were analyzed as described in previous studies.^14^, ^15^

Tumor mutational burden (TMB), immunogenic mutations, copy number, and loss of heterozygosity (LOH) data were all downloaded from the published studies on TCGA cohort.^16^, ^17^, ^18^, ^19^

Gene set enrichment analysis (GSEA) version 2.2.3 was used in this analysis. The corresponding gene set was obtained from the MsigDB database on the GSEA website as the reference gene set, and the enrichment analysis was performed according to the default weighted enrichment statistics method. In this study, the number of random combinations was set to 1000.

### 2.2 Statistical analysis

Overall survival (OS) was defined as the time interval from the date of diagnosis to the date of death. The survival curve was generated by the Kaplan–Meier method, and the difference was compared by the log‐rank test. The restricted mean survival time (RMST)^20^, ^21^ was used to distinguish the 5‐year survival of TFB high and TFB low groups. Differences between TFB high and low groups were tested by the Wilcoxon test or Fisher's exact test (two‐sided). Statistical analysis was performed using SPSS 22.0 version statistical software and R 3.6.1 version. Bilateral *p* < 0.05 was considered statistically significant.

## 3 RESULTS

### 3.1 Cohort characteristics of TFB distribution

**FIGURE 1:**
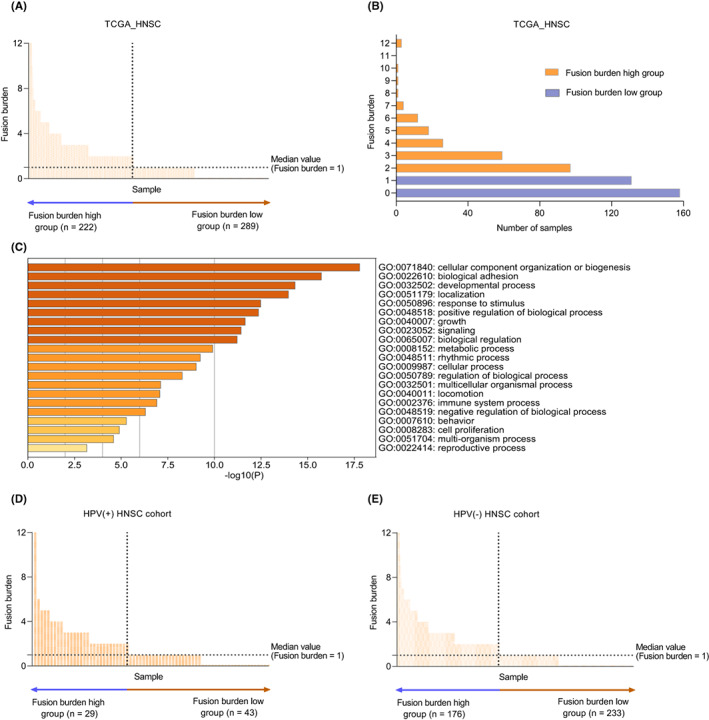
Distribution of tumor fusion burden in the TCGA‐HNSC cohort. (A, B) Distribution of tumor fusion burden in all TCGA_HNSC tumors (*n* = 511). (C) Enrichment analysis of the fusion genes. (D, E) Distribution of tumor fusion burden in the HPV (+) TCGA_HNSC (*n* = 72) or HPV (−) TCGA_HNSC tumors (*n* = 409). TCGA, The Cancer Genome Atlas, HNSC, Head and Neck Squamous Cell Carcinomas, HPV, Human Papillomavirus.

A total of 511 HNSC patients were included in the TCGA‐HNSC data set. As shown in Figure 1A,B, the TFB range of the patients was 0–12 with a median of 1. Among the 511 patients, 222 were classified into the TFB high group as having TFB > 1, and 289 were assigned to the low group as having TFB ≤ 1. The characteristics of the two groups of patients were summarized in Table S1. We next performed gene ontology (GO) enrichment analysis and found that fusion genes were mainly enriched in biological processes such as cellular component organization or biogenesis, biological adhesion, and developmental processes (Figure 1C). In addition, the HNSC patients were divided into HPV (+) group (*n* = 72) and HPV (−) group (*n* = 409) according to their HPV status. The distribution of TFB in the two groups is shown in Figure 1D,E. Thus, TFB = 1, as a cutoff point divided the TFB high group (TFB > 1) and the TFB low group (TFB < =1) used for the subsequent research.

### 3.2 Characteristics of gene variation between TFB high and low groups

**FIGURE 2:**
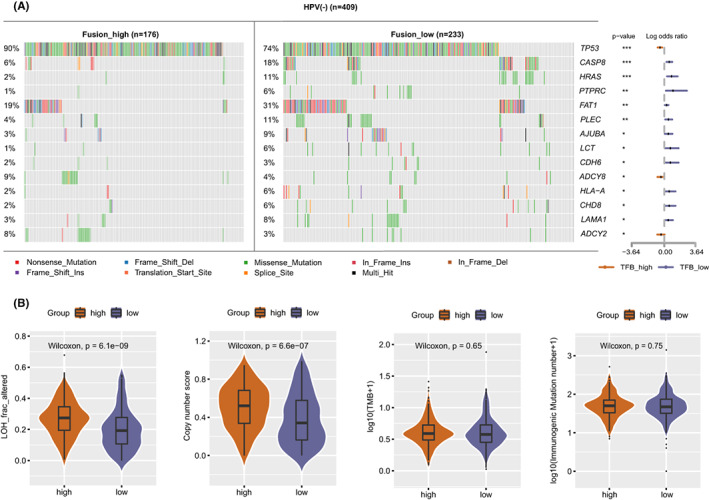
Comparative analysis of the mutational landscape and genomic patterns between TFB high and TFB low HPV (−) HNSC tumors (*n* = 409). (A) Comparison of somatic SNVs between TFB high and TFB low HPV (−) HNSC tumors. Differences were analyzed by Fisher's exact test (two‐sided), *p < 0.05, **p < 0.01, ***p < 0.001. (B) Comparison of LOH fraction, copy number score, TMB, and TNB between TFB high and TFB low HPV (−) HNSC tumors. Differences were analyzed by the Wilcoxon test. TFB, Tumor Fusion Burden, HNSC, Head and Neck Squamous Cell Carcinomas, HPV, Human Papillomavirus, SNV, Single‐Nucleotide Variation, LOH, Loss of Heterozygosity, TMB, Tumor Mutation Burden, TNB, Tumor Neoantigen Burden.

We further analyzed the SNVs of TFB high and low groups to determine whether TFB was damaged by other mutation types. The gene mutation map of HPV (−) and HPV (+) patients in the HNSC cohort was depicted in Figure S1. Among HPV (−) patients, the most frequently mutated gene was TP53, followed by TTN and FAT1. Meanwhile, the TTN gene was the most frequently mutated gene among HPV (+) patients, followed by PIK3CA and MUC16. In the HPV (−) HNSC cohort, TFB high patients had a higher mutation frequency of TP53 gene as well as higher gene copy number and LOH score than TFB low patients (Figures 2A,B and S2A), whereas there were no significant differences in TMB and immunogen mutation numbers between the two groups of patients (Figure 2B). We further explored the effect of TMB in HPV (−) HNSC tumors. The results showed that TMB high cases had a higher mutation frequency of TP53 gene, LOH score, gene copy number, and immunogen mutation numbers in the HPV (−) HNSC cohort (Figure S2B). As shown in Figure S2C,D, no significant differences in SNV sites, LOH, copy number, TMB, and immunogen mutation number were detected between TFB high and TFB low groups in the HPV (+) HNSC cohort.

### 3.3 Relationship between TFB and biological processes in the HPV (−) cohort

**FIGURE 3:**
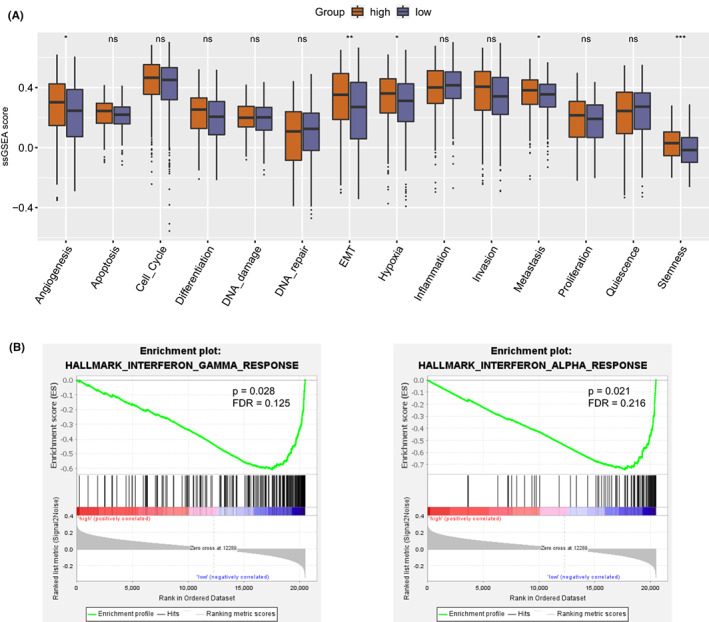
Comparison of the biological processes between TFB high and TFB low HPV (−) HNSC cohorts (*n* = 409). (A) The computational method ssGSEA was applied to compare 14 biological processes including angiogenesis, apoptosis, cell_cycle, differentiation, DNA_damage, DNA_repair, EMT, hypoxia, inflammation, invasion, metastasis, proliferation, quiescence, stemness between TFB high and TFB low HPV (−) HNSC cohorts. Differences were analyzed by the Wilcoxon test, *p < 0.05, **p < 0.01, ns, the difference is not statistically significant. (B) The computational method GSEA was applied to estimate the pathway enrichment in the HPV (−) TCGA‐HNSC cohort. TFB, Tumor Fusion Burden, HNSC, Head and Neck Squamous Cell Carcinomas, HPV, Human Papillomavirus, EMT, Epithelial‐Mesenchymal Transition, TCGA, The Cancer Genome Atlas.

Next, GSEA analysis was carried out to investigate the biological role of TFB in the HPV (−) HNSC. The analysis revealed that in the HPV (−) HNSC cohort, patients in the TFB high group were positively correlated with the biological progress of epithelial‐mesenchymal transition (EMT), metastasis, angiogenesis, and stem cell characteristics (Figure 3A), whereas those in the TFB low group were enriched in IFN‐γ responsive pathway (Figure 3B). Meanwhile, we found that TMB was negatively correlated with angiogenesis, EMT, and metastasis and positively related to cell cycle, differentiation, DNA damage, and DNA repair (Figure S3A).

### 3.4 Relationship between TFB and immune activity in HPV (−) cohort

**FIGURE 4:**
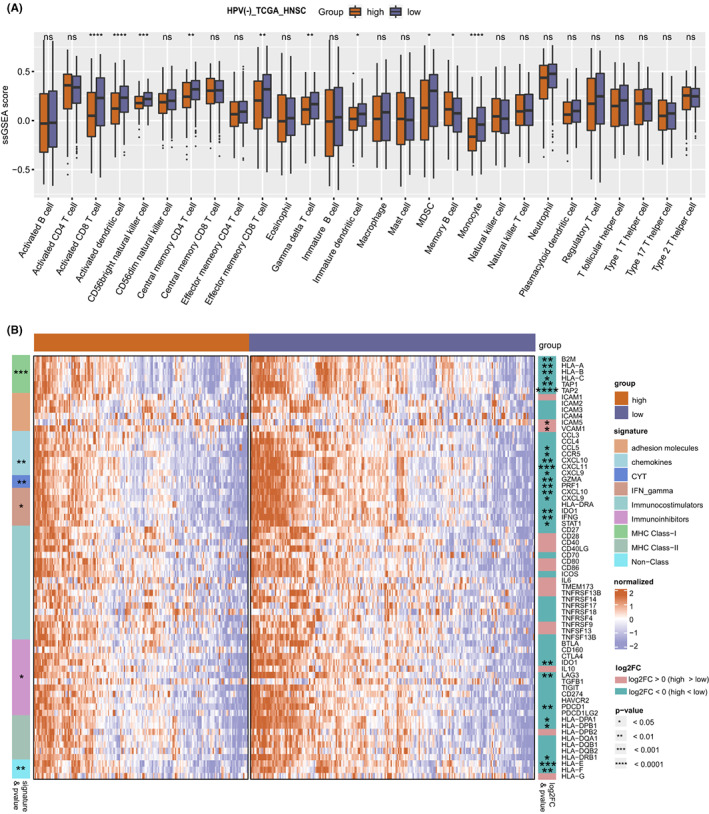
Comparative immune activity analysis between TFB high and TFB low groups in the HPV (−) HNSC cohort (*n* = 409). (A) Boxplot graph showing the comparison of immune cell infiltration between TFB low and high groups. (B) Heatmap depicting the immune‐related signature genes expression in TFB low tumors compared with TFB high tumors. Differences were analyzed by the Wilcoxon test, *p < 0.05, **p < 0.01, ***p < 0.001, ****p < 0.0001. TFB, Tumor Fusion Burden, HNSC, Head and Neck Squamous Cell Carcinomas, HPV, Human Papillomavirus.

Given the significant enrichment of IFN‐γ‐responsive gene sets in TFB low tumors, we further investigate the difference in immune activity between TFB high and low tumors. We estimated the abundance of 15 kinds of acquired immune cells and 13 kinds of innate immune cells. These 28 kinds of immune cells include most types of lymphocytes which produce important cytokines. The ssGSEA analysis revealed that the infiltration of immune cells in the HPV (+) HNSC cohort was higher than that in the HPV (−) HNSC cohort (Figure S4A). In the entire cohort, patients in the TFB low group displayed an increased immune cell infiltration compared with the TFB high group (data not shown). Notably, HPV (−) tumors with low TFB were more infiltrated by the activated CD8 T cell, activated dendritic cell, CD56 bright natural killer cell, and effector memory CD8 T cell compared with HPV (−) tumors with high TFB (Figure 4A), no significant difference in the immune cell infiltration was observed between TFB low and TFB high groups in the HPV (+) HNSC cohort (*p* > 0.05, data not shown). We further found that TMB was positively correlated to active CD4 T cell and negatively related to central memory CD4 T cell, gamma delta T cell, monocyte, regulatory T cell, and type 1, type 2, and type17 T helper cells (Figure S4B). In addition, HPV (−) patients with low TFB exhibited significantly stronger cytolytic activity and elevated expression levels of chemokine signature genes, immunoinhibitors, MHC‐I genes, chemokines, and interferon‐gamma (IFN‐γ) signature genes than those with high TFB (Figure 4B).

### 3.5 The relationship between TFB and prognosis in HPV (−) cohort

**FIGURE 5:**
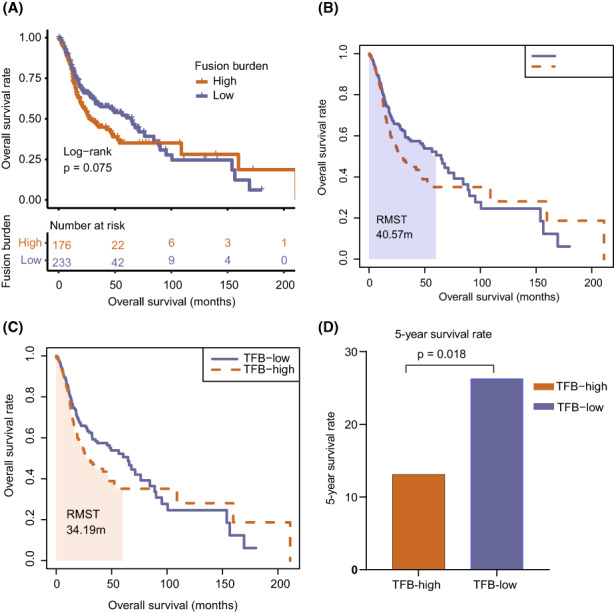
Kaplan–Meier analysis of overall survival in HPV (−) HNSC cohort. (A) Comparison of overall survival among patients with high and low TFB in the HPV (−) HNSC cohort (*n* = 409, log‐rank test, *p* = 0.075). (B, C) The area under the Kaplan–Meier curves within the window of 60 months (5 years) for TFB low and TFB high groups (*n* = 409). (D) Comparison of 5‐year survival rate among patients with high and low TFB in the HPV (−) HNSC cohort (*n* = 217). TFB, Tumor Fusion Burden, HNSC, Head and Neck Squamous Cell Carcinomas, HPV, Human Papillomavirus.

To determine whether TFB exerts a significant effect on the prognosis of HPV (−) HNSC patients, we conducted a survival analysis on the cohort of TFB high and TFB low patients. In the HPV (−) HNSC cohort, patients with low TFB had a longer OS than those with high TFB (Figure 5A). Moreover, the RMST^20^, ^21^ was used to distinguish the survival of TFB high and TFB low groups. Five‐year survival profile was the area under the Kaplan–Meier curves from months 0 to 60 (TFB low group, shaded areas in Figure 5B; TFB high group, shaded areas in Figure 5C). The data demonstrated that patients with the TFB low group live a mean of 40.57 months in 5 years, which is 6.38 months (95% CI, 0.74–0.95; *p* = 0.013, data not shown) longer than survival among TFB high patients (34.19 months).

A total of 217 HPV (−) HNSC patients died within 5 years or survived for more than 5 years (173 patients died within 5 years, and 44 patients survived for more than 5 years), the other 192 patients were still alive and followed‐up for <5 years. Among the 217 patients, 31 patients in the TFB low group (*n* = 118) and 13 patients in the TFB high group (*n* = 99) survived more than 5 years. The 5‐year survival rates in the TFB low and high groups were 26.4% and 13.1% respectively. The 5‐year survival rate of patients with the TFB low group was significantly higher than that of the TFB high group (*p* = 0.018, Figure 5D).

In addition, we also explored the characteristics and clinical relevance of TFB in HNSC subsites, including oral cavity, oropharynx, and larynx cancers (Figures S5–S8). In the HPV (−) oral cavity (Figure S5), oropharynx (Figure S6), and larynx patients (Figure S8), the results were consistent with the total HPV (−) HNSC patients. In the HPV (+) oropharynx patients, no significant relations were found between TFB and tumor immune microenvironment, genomic patterns, and survival time (Figure S7).

In conclusion, our data revealed that HPV (−) HNSC patients with low TFB exhibited stronger immune activity, lower LOH, and gene copy number score, indicating TFB may be a potential immunotherapeutic marker. Moreover, low TFB was related to prolonged OS in the HPV (−) HNSC cohort.

## 4 DISCUSSION

The pathogenesis of HNSC, the most common malignant tumor in the head and neck, involves multiple factors such as genetic variation, epigenetics, and environment, while it is prone to early local infiltration and lymph node metastasis.^22^, ^23^ The prognosis of HNSC patients remains poor, and it is an urgent need to identify biomarkers and drug treatment targets with effective predictive power.^1^ With the tremendous advances in high‐throughput technologies, such as microarrays and RNA sequencing, gene mapping has become a powerful tool for understanding cancer progression, determining treatment options, and identifying molecular biomarkers predicting patient outcomes.^2^, ^24^ Gene fusion represents an important type of somatic changes in cancer. Highly recurrent fusions have been found in prostate cancer, bladder cancer, breast cancer, and lung cancer.^13^ In this study, we applied multiple RNA‐Seq fusion callers and screened 222 cases of the TFB high group as well as 289 cases of the TFB low group. The TFB of the 511 cases was in the range of 0–12, which was lower than that in prostate cancer,^9^ but consistent with the report of Gao et al.^13^ In this case, the number of fusion genes in prostate cancer was higher than that in HNSC patients. Furthermore, we investigated the prognosis of HNSC patients with high or low TFB and the relationship of the prognosis with immune activity from immune infiltration of tumor microenvironment of HPV (−)‐related HNSC. The results showed that patients with high TFB harbored higher mutation frequency of TP53 gene, gene copy number, and LOH score than those with low TFB, while they were positively correlated with the biological progress of EMT, metastasis, angiogenesis, and stem cell characteristics. In addition, patients in the TFB low group were mainly enriched in the IFN‐γ responsive pathway. Further analysis revealed that patients with low TFB in the HPV (−) HNSC cohort have higher immune scores and have a better prognosis.

According to reports, one of the etiological pathways of HNSC is through HPV.^4^ There are genetic and prognostic differences between HPV (+) and HPV (−) HNSC. The heterogeneity of this cancer suggests a critical role for genetic alterations contributing to its carcinogenesis.^25^ It has been shown that mutation in TP53 is the most common somatic mutation in head and neck cancer and can be frequently detected in HPV(−) tumors.^26^ The present study found that among patients in the TFB high group, those with HPV (−) had a higher mutation frequency of TP53, gene copy number, and LOH scores. In HPV (+) patients, there were no significant differences in gene copy number and LOH score between TFB high and TFB low groups. The above observations are consistent with the previous findings. TP53 mutations are rarely detected in HPV‐positive tumors due to the fact that E6 oncoprotein in HPV mediates ubiquitination and proteasome degradation of p53.^27^ Guo et al.^28^ reported that TP53 mutation can induce an inhibitory immune microenvironment and promote tumor immune evasion. Instable copy number and LOH may also lead to an immune evasion, which is a characteristic feature of cancer and is involved in the mechanism underlying acquired resistance to immunotherapy.^18^

Previous studies have shown that tumors with fusion events tend to have decreased TMB.^9^ Conversely, we observed in this study that there was no significant difference in TMB between HNSC patients with low TFB and those with high TFB. It has been demonstrated that there were interspecies differences of TMB in malignant tumors. In this case, multiple studies on TFB in the tumor microenvironment found that prostate cancer patients with high TFB tend to have elevated immune infiltration.^9^ Another study showed that T cell effector factors are more abundant in colorectal cancer with high immune infiltration, and the secretion of cytokine IFN‐γ is increased, leading to tumor cell lysis and apoptosis.^29^ In the present study, we obtained consistent data with the previous studies on colorectal cancer, showing that patients in the TFB low group have elevated immune cell infiltration compared with those in the TFB high group, and the immune signatures are highly enriched.

Molecular analysis of HNSCs has identified significant correlations of specific immune cell subpopulations' infiltration with distinct gene expression patterns such as HPV and genetic alterations as well as with survival.^30^ A previous study found that the prognosis of colorectal cancer patients with high immune infiltration was significantly better than that of the patients with low immune infiltration.^31^ Another study showed that higher CD8+ T‐cell infiltration has been observed among anti‐PD1/L1 therapy responders and has been proven to be an independent predictive factor for improved prognosis.^32^ Gene fusion is also linked with prognosis of cancer patients. In a study on gastric cancer, the fusion gene promotes EMT and enhances the ability of tumor cells to metastasize, thereby affecting the prognosis.^33^ Similar to these studies, the results showed that patients with high TFB harbored higher mutation frequency of TP53 gene, gene copy number, and LOH score than those with low TFB, while they were positively correlated with the biological progress of EMT, metastasis, angiogenesis, and stem cell characteristics. Moreover, we analyzed the survival of the HPV (−) HNSC patients with regard to the TFB level and found that compared with the patients with high TFB, those with low TFB exhibit a better prognosis as well as a significant increase in the OS and 5‐year survival rate. The above observations were consistent with the previous studies on immune activity and SNVs. Similarly, consistent with a study focused on squamous cell carcinomas by Li et al.^34^ immune cell infiltration is more abundant, IFN‐γ response and cytolytic activity levels are higher, it is a kind of “immune‐hot tumor” and is related to a good prognosis.

Taken together, patients with TFB low in the HPV (−) HNSC cohort have enhanced immune activity and higher levels of predictive factors for immunotherapy, suggesting patients with low TFB may be potential candidates for immunotherapeutic agents. Importantly, the clinical analysis revealed that tumors with low TFB had prolonged OS in the HPV (−) HNSC cohort.

## AUTHORS' CONTRIBUTION

Lirui He, Dandan Ren, Longlong Gong, and Ping Liu designed the research. Lirui He and Dandan Ren wrote the manuscript and analyzed the data. Guoqing Lv, Beibei Mao, Lijia Wu, and Xiaoyu Liu collected the data and technical information. Longlong Gong and Ping Liu reviewed and helped revise the manuscript. All authors approved the final proof.

## FUNDING INFORMATION

The authors wish to acknowledge the financial support from Beijing Xisike Clinical Oncology Research Foundation (Y‐sy2018‐145) and the Shenzhen Sanming Project (Grant No. SZSM201612051).

## CONFLICT OF INTEREST

The authors made no disclosures.

## ETHICAL DISCLOSURE

All data were downloaded from the TCGA (TCGA, PanCancer Atlas) via the cBioPortal (http://www.cbioportal.org/). TCGA belongs to public databases. The patients involved in the database have obtained ethical approval. Users can download relevant data for free for research and publish relevant articles. Our study is based on open‐source data, so there are no ethical issues and other conflicts of interest.

## Supporting information


Table S1
Figure S1‐S4

Figure S5‐S8

## Data Availability

The data that support the findings of this study are available from the corresponding author and literature upon reasonable request.
