# Supplementary material for: The characteristics and clinical relevance of tumor fusion burden in head and neck squamous cell carcinoma
Source: Cancer Med. 2022 May 27;12(1):852–61. doi: 10.1002/cam4.4890 (PMC9844600; doi:10.1002/cam4.4890)
Supplement: Supplementary file 1 — Table S1 Figure S1‐S4 [file CAM4-12-852-s001.docx]

**Supplement materials**

Table S1 The clinical features between fusion burden high and low groups in the TCGA_HNSC cohort

| **Factor** | **Overall (n=511)** | **Fusion burden high (n=222)** | **Fusion burden low (n=289)** | ***P* value** |
| --- | --- | --- | --- | --- |
| **Age** (years) |  |  |  | 0.787 |
| median | 61.00 | 61.00 | 60.00 |  |
| IQR | 53.00-68.00 | 53.00-68.00 | 53.00-69.00 |  |
| **Gender** (%) |  |  |  | 0.61 |
| Female | 131 (25.6) | 54 (24.3) | 77 (26.6) |  |
| Male | 380 (74.4) | 168 (75.7) | 212 (73.4) |  |
| **Primary site** (%) |  |  |  | 0.09 |
| Oropharynx | 79 (15.5) | 40 (18.0) | 39 (13.5) |  |
| Hypopharynx | 10 (2.0) | 4 (1.8) | 6 (2.1) |  |
| Larynx | 115 (22.5) | 58 (26.1) | 57 (19.7) |  |
| Oral cavity | 307 (60.1) | 120 (54.1) | 187 (64.7) |  |
| **HPV** **infection** (%) |  |  |  | 0.7 |
| Negative | 409 (80.0) | 176 (79.3) | 233 (80.6) |  |
| Positive | 72 (14.1) | 29 (13.1) | 43 (14.9) |  |
| NA | 30 (5.9) | 17 (7.7) | 13 (4.5) |  |
| **RACE** (%) |  |  |  | 0.071 |
| American Indian or Alaska Native | 2 (0.4) | 1 (0.5) | 1 (0.3) |  |
| Asian | 11 (2.2) | 8 (3.6) | 3 (1.0) |  |
| Black or African American | 47 (9.2) | 25 (11.3) | 22 (7.6) |  |
| White | 436 (85.3) | 182 (82.0) | 254 (87.9) |  |
| NA | 15 (2.9) | 6 (2.7) | 9 (3.1) |  |
| **pStage** (%) |  |  |  | 0.76 |
| Stage I | 24 (4.7) | 9 (4.1) | 15 (5.2) |  |
| stage II | 75 (14.7) | 29 (13.1) | 46 (15.9) |  |
| stage III | 78 (15.3) | 32 (14.4) | 46 (15.9) |  |
| stage IV | 265 (51.9) | 118 (53.2) | 147 (50.9) |  |
| NA | 69 (13.5) | 34 (15.3) | 35 (12.1) |  |
| **pTstage** (%) |  |  |  | 0.68 |
| T0 | 1 (0.2) | 1 (0.5) | 0 (0.0) |  |
| T1 | 45 (8.8) | 19 (8.6) | 26 (9.0) |  |
| T2 | 134 (26.2) | 58 (26.1) | 76 (26.3) |  |
| T3 | 96 (18.8) | 39 (17.6) | 57 (19.7) |  |
| T4 | 173 (33.9) | 76 (34.2) | 97 (33.6) |  |
| TX | 39 (7.6) | 21 (9.5) | 18 (6.2) |  |
| NA | 23 (4.5) | 8 (3.6) | 15 (5.2) |  |
| **pNstage** (%) |  |  |  | 0.151 |
| N0 | 170 (33.3) | 63 (28.4) | 107 (37.0) |  |
| N1 | 67 (13.1) | 28 (12.6) | 39 (13.5) |  |
| N2 | 167 (32.7) | 84 (37.8) | 83 (28.7) |  |
| N3 | 9 (1.8) | 5 (2.3) | 4 (1.4) |  |
| NX | 73 (14.3) | 32 (14.4) | 41 (14.2) |  |
| NA | 25 (4.9) | 10 (4.5) | 15 (5.2) |  |
| **pMstage** (%) |  |  |  | 0.579 |
| M0 | 186 (36.4) | 76 (34.2) | 110 (38.1) |  |
| M1 | 1 (0.2) | 1 (0.5) | 0 (0.0) |  |
| MX | 62 (12.1) | 24 (10.8) | 38 (13.1) |  |
| NA | 262 (51.3) | 121 (54.5) | 141 (48.8) |  |
| **ETHNICITY** (%) |  |  |  | 0.835 |
| Hispanic Or Latino | 24 (4.7) | 11 (5.0) | 13 (4.5) |  |
| Not Hispanic Or Latino | 450 (88.1) | 195 (87.8) | 255 (88.2) |  |
| NA | 37 (7.2) | 16 (7.2) | 21 (7.3) |  |

***P* value**: Wilcoxon test rank sum or Fisher’s exact test (two sided) was used for the comparison between the fusion burden high and low groups.

NA: not available.

HNSC, Head and Neck Squamous Cell Carcinomas, TCGA, The Cancer Genome Atlas.

Figure S1 Mutational landscape of TFB-high and TFB-low tumors in the TCGA HNSC cohort (n=481). (A) HPV (-) TCGA_HNSC(n=409). (B) HPV (+) TCGA_HNSC(n=72). TFB, Tumor Fusion Burden, HNSC, Head and Neck Squamous Cell Carcinomas, HPV, Human Papillomavirus, TCGA, The Cancer Genome Atlas.

Figure S2 Comparative analysis of the mutational landscape and genomic patterns. (A) Comparison of TP53 mutation status between TFB high and low HPV (-) HNSC tumors. (B) Comparison of TP53 mutation status, LOH fraction, copy number score and immunogen mutation numbers between TMB high and low HPV (-) HNSC tumors. (C) Comparison of somatic SNVs between TFB high- and TFB low HPV (+) HNSC tumors (n=72). (D) Comparison of LOH fraction, copy number score, TMB and TNB between TFB high- and TFB low HPV (+) HNSC tumors. Differences were analyzed by Wilcoxon test, ****P*<0.001, *****P*<0.0001. TFB, Tumor Fusion Burden, TMB, Tumor Mutation Burden, LOH, Loss of Heterozygosity, HNSC, Head and Neck Squamous Cell Carcinomas, HPV, Human Papillomavirus, TCGA, The Cancer Genome Atlas.

Figure S3 Comparison of the biological processes between TMB high- and TMB low HPV (-) HNSC cohort. (A) The computational method ssGSEA was applied to compare 14 biological processes including angiogenesis, apoptosis, cell_cycle, differentiation, DNA_damage, DNA_repair, EMT, hypoxia, inflammation, invasion, metastasis, proliferation, quiescence, stemness between TMB high- and TMB low HPV (-) HNSC cohort. Differences were analyzed by Wilcoxon test, **P*<0.05, ***P*<0.01, ****P*<0.001, *****P*<0.0001, ns, the difference is not statistically significant. TMB, Tumor Mutation Burden, HNSC, Head and Neck Squamous Cell Carcinomas, HPV, Human Papillomavirus, EMT, Epithelial-Mesenchymal Transition, TCGA, The Cancer Genome Atlas.

Figure S4 Analysis of immune infiltration in the TCGA HNSC cohort. (A) The computational method ssGSEA was applied to estimate the abundance of 28 kinds of immune cells including 15 kinds of adaptive immune cells and 13 kinds of innate immune cells in HPV (-) and HPV (+) TCGA_HNSC cohort (n=481). (B) The computational method ssGSEA was applied to estimate the abundance of 28 kinds of immune cells including 15 kinds of adaptive immune cells and 13 kinds of innate immune cells between TMB high- and TMB low group in the HPV (-) HNSC cohort (n=409). Differences were analyzed by Wilcoxon test, **P*<0.05, ***P*<0.01, ****P*<0.001, *****P*<0.0001, ns, the difference is not statistically significant. TFB, Tumor Fusion Burden, TMB, Tumor Mutation Burden, HNSC, Head and Neck Squamous Cell Carcinomas, HPV, Human Papillomavirus, TCGA, The Cancer Genome Atlas.
