# Supplementary material for: The characteristics and clinical relevance of tumor fusion burden in head and neck squamous cell carcinoma
Source: Cancer Med. 2022 May 27;12(1):852–61. doi: 10.1002/cam4.4890 (PMC9844600; doi:10.1002/cam4.4890)
Supplement: Supplementary file 2 — Figure S5‐S8 [file CAM4-12-852-s002.pdf]

Figures S5-8

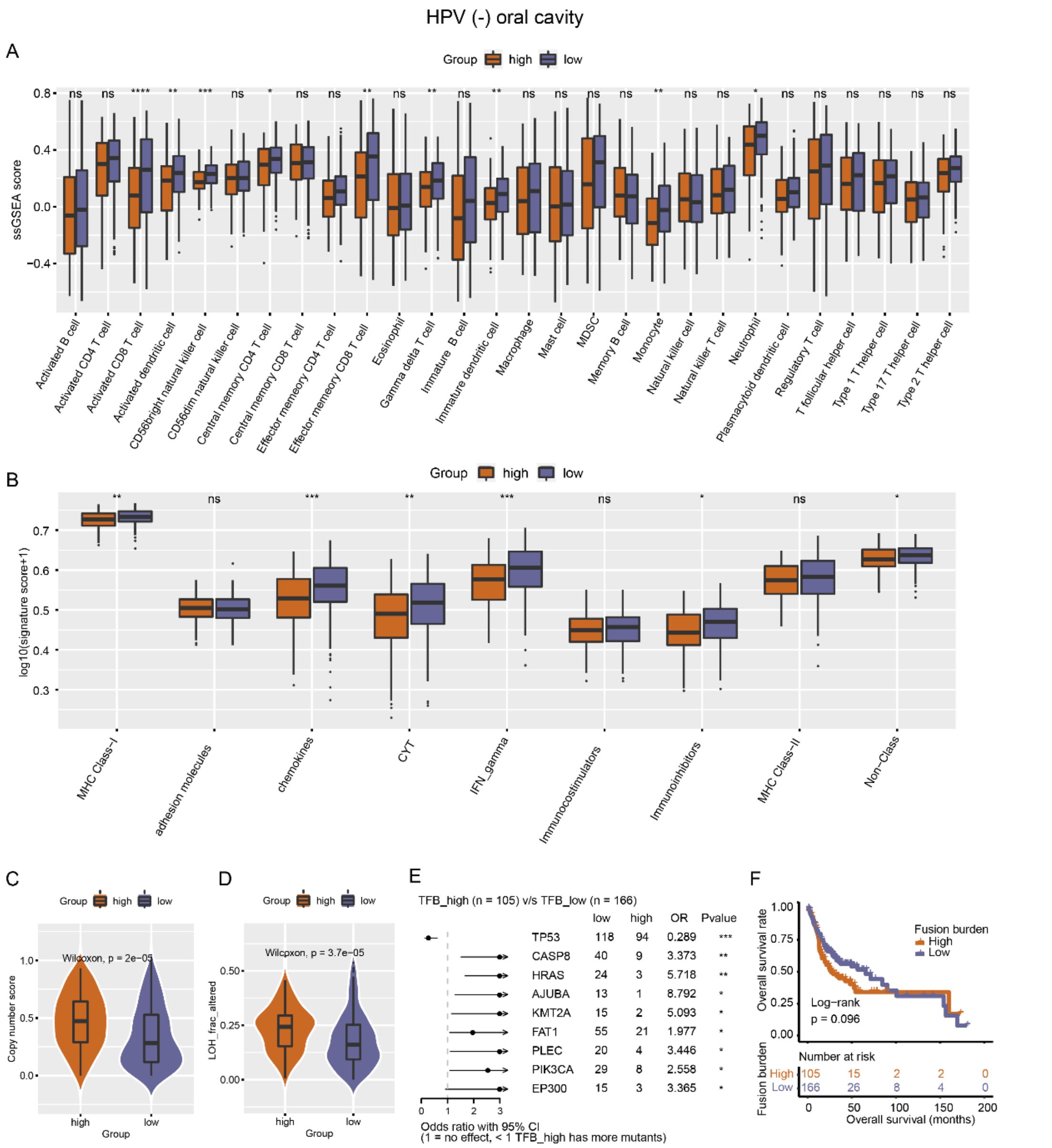

Figure S5. Correlation of TFB with patients with (A) immune cells infiltration, (B) expression level of immune features, score of (C) gene copy number and (D) LOH, (E) gene mutation, and (F) OS in HPV (-) oral cavity patients.

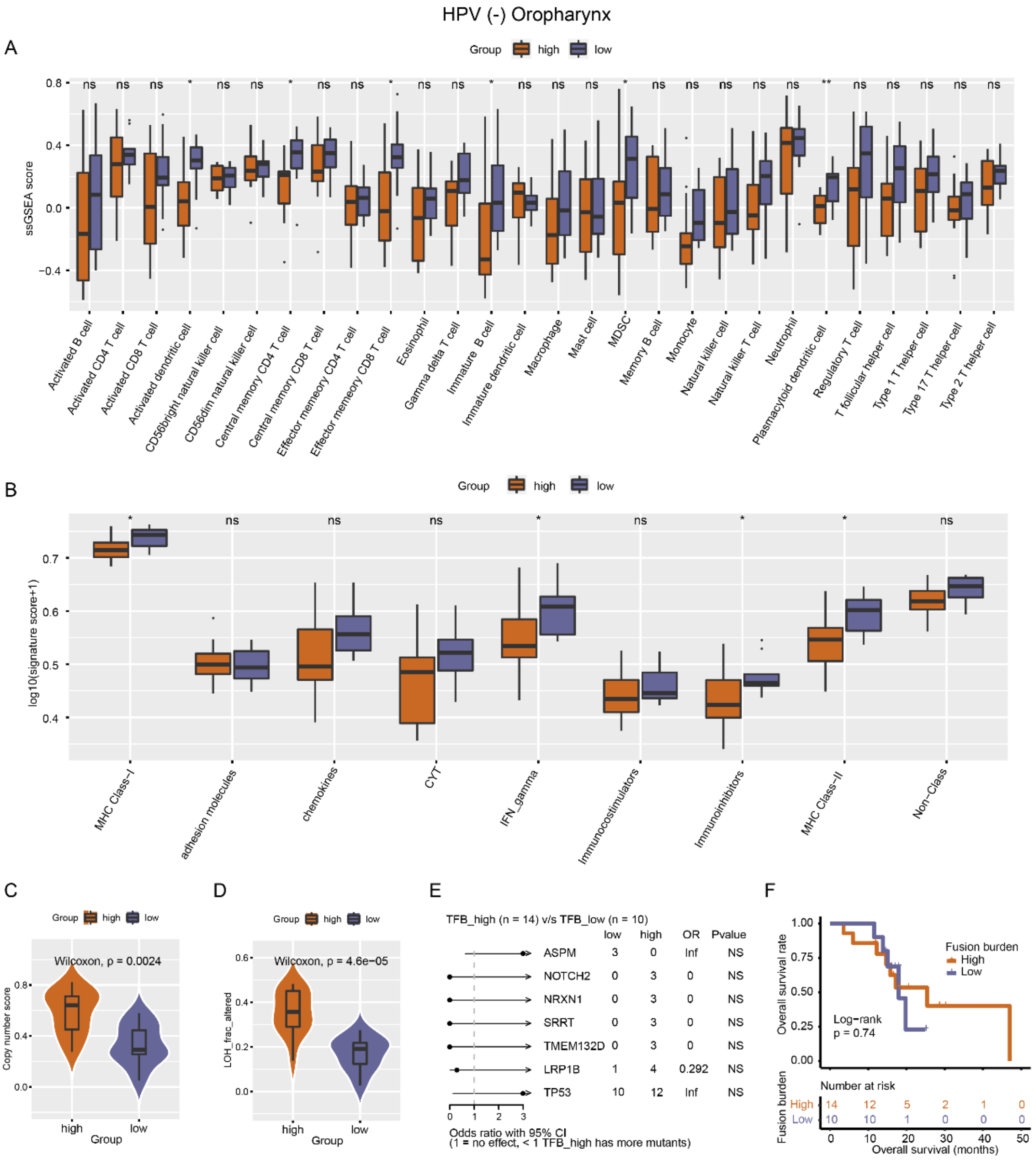

Figure S6. Correlation of TFB with patients with (A) immune cells infiltration, (B) expression level of immune features, score of (C) gene copy number and (D) LOH, (E) gene mutation, and (F) OS in HPV (-) oropharynx patients.

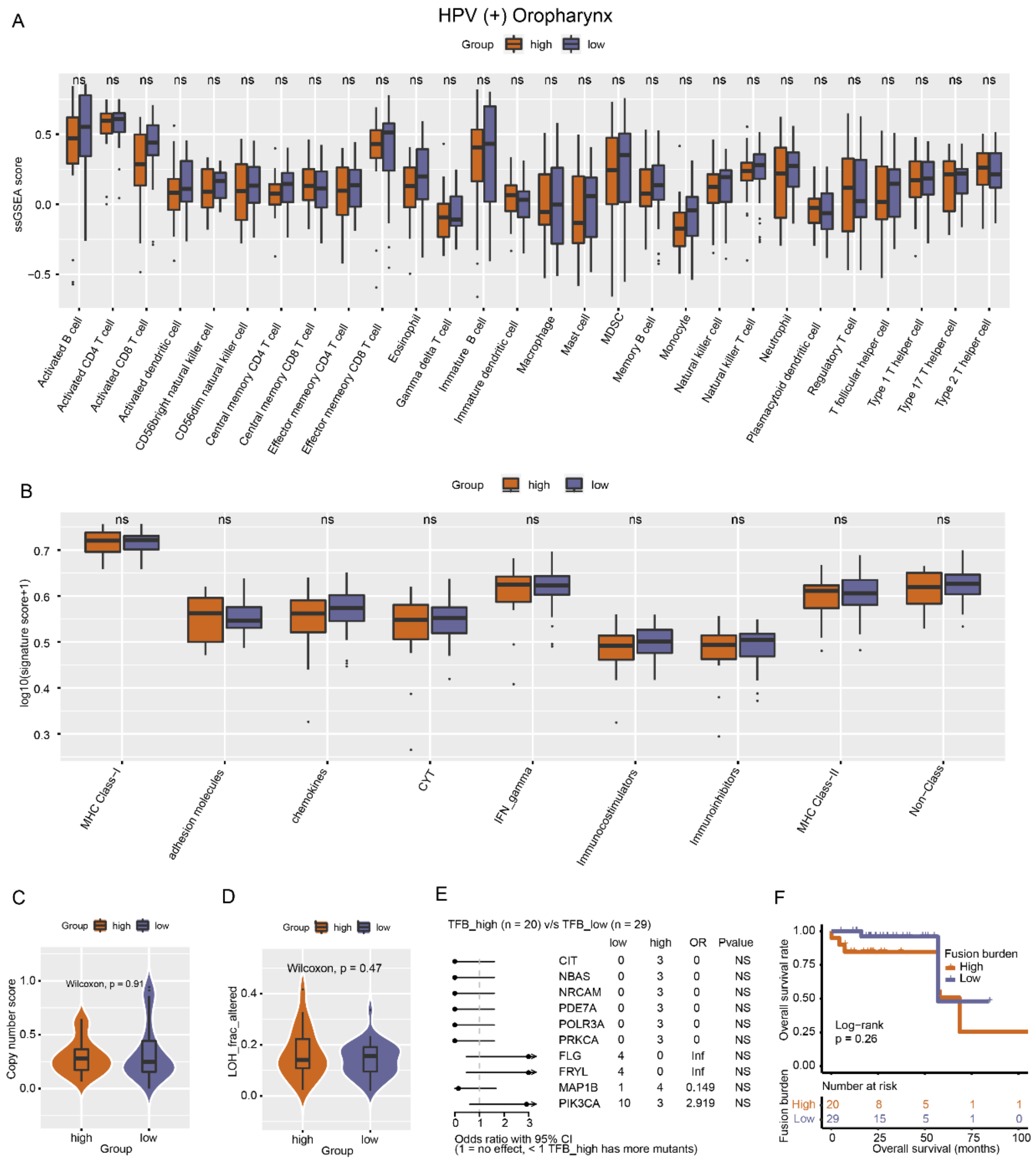

Figure S7. Correlation of TFB with patients with (A) immune cells infiltration, (B) expression level of immune features, score of (C) gene copy number and (D) LOH, (E) gene mutation, and (F) OS in HPV (+) oropharynx patients.

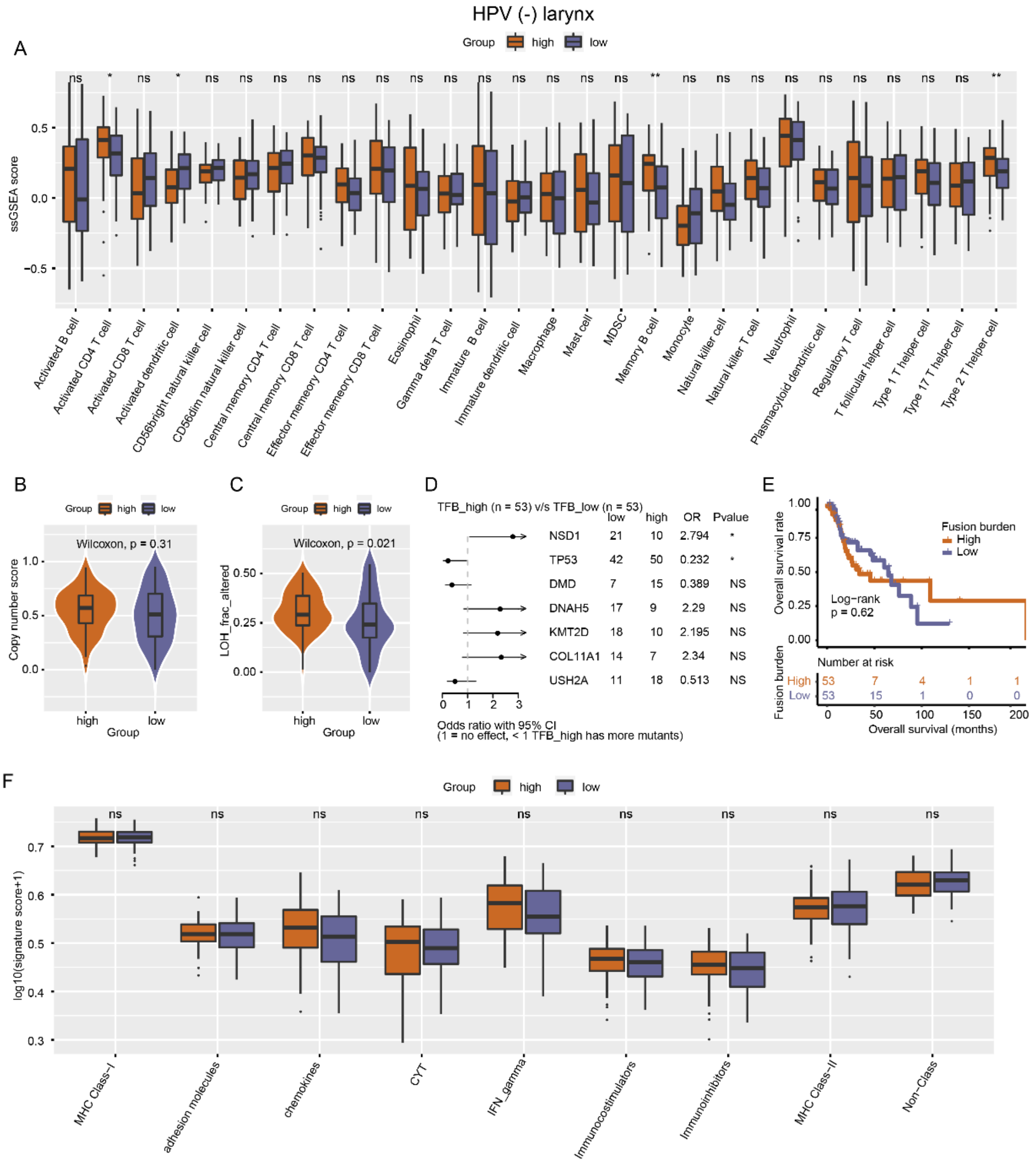

Figure S8. Correlation of TFB with patients with (A) immune cells infiltration, score of (B) gene copy number and (C) LOH, (D) gene mutation, and (E) OS, and (F) expression level of immune features, in HPV (-) larynx patients.
